# Astrocyte–Neuron Crosstalk in Hypertension: Mechanisms and Therapeutic Significance

**DOI:** 10.31083/RCM47764

**Published:** 2026-04-14

**Authors:** Mengyu Zhao, Huizhuo Jia, Na Li

**Affiliations:** ^1^School of Basic Medical Sciences, Hebei University, 071000 Baoding, Hebei, China; ^2^Key Laboratory of Aging and Health in Hebei Province, 071000 Baoding, Hebei, China

**Keywords:** hypertension, astrocytes, neuron, central nervous system

## Abstract

Hypertension is a major global health challenge that poses a serious threat to cardiovascular function. Crosstalk between astrocytes and neurons plays a central role in blood pressure regulation within the central nervous system. As the prevalence of hypertension continues to increase, significant progress has been made in understanding the associated pathological mechanisms involving astrocytes and neurons. Accumulating evidence indicates that astrocytes engage in complex metabolic–immune networks to communicate with neurons, thereby contributing critically to the development and progression of hypertension. This review highlights recent advances in our understanding of the bidirectional regulatory mechanisms between astrocytes and neurons in the context of hypertension.

## 1. Introduction

Hypertension is a principal risk factor for cardiovascular and cerebrovascular events, renal failure, and cognitive impairment. Over the past 30 years, the number of adults worldwide affected by hypertension has nearly doubled, and approximately 8 million deaths worldwide each year are attributable to hypertension-related cardiovascular disease [1, 2]. A growing body of clinical and preclinical data suggests that the central nervous system (CNS), specifically the dysregulated central control of sympathetic outflow, is a key factor in the onset and progression of hypertension, even though traditional research has focused on peripheral mechanisms like vascular remodeling, the renin-angiotensin system, and systemic vascular resistance [3, 4, 5]. This research model shift from a predominantly “vascular” perspective toward a “brain-controlled” model of hypertension has catalyzed new mechanistic studies and therapeutic concepts.

Research on hypertension has revealed a strong link between astrocytes and the pathophysiology of the CNS. About 20–50% of CNS cells are astrocytes, the biggest glial population in the mammalian CNS, and they are closely associated with a variety of neurological conditions [6]. Mature astrocytes display remarkable morphological, transcriptional, functional, and phenotypic heterogeneity: their shape, signaling modalities, and roles vary across brain regions and neural circuits, and they are critical for CNS development, homeostatic adaptation, and aging [7]. Neuronal activity depends on dynamic energy supply, yet neurons themselves have minimal intrinsic energy stores; consequently, neuronal function relies heavily on astrocytic metabolic support [8]. Astrocytes mediate glucose uptake via glucose transporter 1 (GLUT1) and shuttle energy substrates to neurons through monocarboxylate transporters (MCT1/4), sustaining synaptic transmission and firing patterns. This cooperative pathway is commonly referred to as the astrocyte–neuron lactate shuttle (ANLS) [9, 10]. Additionally, astrocytes regulate calcium signals, neuro-vascular coupling, and K^+^ buffering, among other mechanisms, to maintain a balance between brain metabolism and neural excitability [11, 12]. The in-depth study of astrocytes has revealed that they are closely associated with neurons and play a significant regulatory function in hypertension, thanks to the ongoing advancements in physiology, anatomy, proteomics techniques, and single-cell transcriptomics technologies.

Significant changes in astrocyte shape and metabolic activity are correlated with hypertension. For instance, astrocytic endfeet retract and aquaporin 4 (AQP4) expression is downregulated in the spontaneously hypertensive rat model, suggesting impaired neuroglial support [13]. Meanwhile, angiotensin II (Ang II) and inflammatory factors (such as interleukin-1 beta (IL-1β)) can induce metabolic reprogramming in astrocytes, driving their polarization from a supportive phenotype toward an excitatory or pro-inflammatory phenotype [14]. These astrocytic changes are concentrated within central autonomic hubs that regulate blood pressure, for example, the paraventricular nucleus (PVN) of the hypothalamus and the rostral ventrolateral medulla (RVLM). Dysfunctional astrocyte–neuron interactions in these regions facilitate excessive sympathetic outflow and increased peripheral vascular tone, contributing to the initiation and maintenance of hypertension.

In this context, astrocyte–neuron metabolic dysregulation is increasingly recognized as a critical node of metabolic imbalance underlying hypertensive pathophysiology. By adopting a multidimensional perspective that integrates metabolic processes, neural regulation, and blood pressure control, research into the dynamic interplay between astrocytes and neurons may not only deepen our understanding of central pathogenic mechanisms in hypertension but also offer a theoretical basis and potential targets for novel centrally acting interventions. This review systematically outlines the physiological foundation of astrocyte-neuron communication, their pathological alterations in hypertension, and discusses emerging therapeutic strategies and future research directions.

### Physiological Functions of Astrocytes

Astrocytes are the most abundant glial cell type in the CNS, arising from neural progenitors in the subventricular zone and radial glia in the ventricular zone. During neurodevelopment, they mature and migrate to populate the entire CNS, where they perform essential roles in neuronal differentiation, synaptogenesis, energy supply, extracellular ion homeostasis, and blood–brain barrier (BBB) integrity [15, 16, 17], performing as versatile modulators of circuit function in both developing and mature brains. Astrocytes exert multifaceted control over neural networks by secreting synaptogenic proteins such as thrombospondins [18], expressing phagocytic receptors like Mertk and Megf10 that mediate synaptic pruning [19], and releasing factors that regulate synapse maturation [20].

In a healthy brain, astrocytes occupy a pivotal position of the neurovascular unit, for which their endfeet closely appose capillaries, enabling high-efficiency glucose uptake via GLUT1 and rapid conversion of glucose to lactate, which is exported through MCT1/MCT4 to fuel neighboring neurons. Simultaneously, synaptically released glutamates are cleared by astrocytic excitatory amino acid transporter (EAAT), triggering Na^+^/K^+^-ATPase activity that drives glycolysis and augments lactate production, completing the process of glutamate–lactate coupling or ANLS [10, 21]. When neuronal firing elevates extracellular K^+^, astrocytes quickly buffer ionic changes via Kir4.1 and Na^+^/K^+^-ATPase, and propagate metabolic and haemodynamic signals across the glial syncytium by intracellular Ca^2+^ waves [22, 23]. Through this exact coordination, astrocytes provide stable metabolic support for neural activities by dynamically distributing energy substrates like glucose, lactic acid, and glutamate to the BBB through their foot processes, in addition to maintaining the immediate adenosine triphosphate (ATP) supply for neurons in a high-energy-consuming state [24]. Given that this crosstalk is crucial for maintaining neuronal function, pathological alterations in astrocytes themselves can readily impair their regulatory capacity over metabolic and ionic homeostasis, thereby giving rise to a distinct pathological scenario in the context of hypertension.

## 2. Pathological Alterations of Astrocytes Under Hypertensive Conditions

### 2.1 Mechanisms of Astrocyte Reactive Gliosis

Under hypertensive conditions, astrocytes undergo marked reactive astrogliosis, manifested by somatic hypertrophy, proliferation, and upregulation of intermediate filament proteins including glial fibrillary acidic protein (GFAP).

In experimental hypertension, astrocyte density and soma volume significantly increase in brainstem autonomic regions, including the nucleus tractus solitarii (NTS) and the RVLM, where these changes coincide with neuroinflammatory markers linked to elevated blood pressure [25].

The tissues of the CNS emit a variety of chemicals, such as ATP, heat shock proteins (HSPs), reactive oxygen species (ROS), norepinephrine (NE), glutamate, etc., under pathological situations like hypertension. These substances further activate astrocytes by leaking into the cerebrospinal fluid and passing through the BBB [26, 27]. In addition, molecules such as IL-1β, interleukin-1 alpha (IL-1α), tumor necrosis factor-alpha (TNF-α), interleukin-6 (IL-6), ciliary neurotrophic factor (CNTF), and transforming growth factor-beta (TGFβ), released by astrocytes themselves, microglia, and other immune cells, can bind to receptors on astrocytes and trigger reactive astrogliosis [28, 29, 30].

High-pressure Overload Shock (HOS) can also induce reactive changes in astrocytes, including the upregulation of the gap junction protein Connexin 43 (Cx43), a key player in ion/metabolic buffering and second messenger communication in physiology and disease [31, 32, 33].

In addition, fluctuations in Ca^2+^ in astrocytes are a fundamental mechanism through which they regulate synaptic activity. Both excessive and insufficient Ca^2+^ signaling can impact central autonomic neurons. ATP, secreted by astrocytes as a key mediator, couples Ca^2+^ signaling to neuronal excitation and cardiovascular control. In the PVN, the ATP released by astrocytes highlights their central role in regulating sympathetic output and blood pressure control [34]. In mice, this ATP release is controlled by calcium-dependent connexin 43 hemichannels in astrocyte gap junctions, leading to the excitation of neural circuits involved in cardiovascular regulation [35]. In the NTS, a major brain region responsible for pressure reflex functions, studies have demonstrated that glutamate and 5-hydroxytryptamine (5-HT) at afferent nerve terminals trigger calcium-dependent ATP release in astrocytes. This ATP, acting on P2Y purinoceptor 1 (P2Y_1_) receptors, finely tunes the sensitivity of pressure reflexes, thereby contributing to blood pressure stability [36]. The ATP signaling mechanism in astrocytes also contributes to the RVLM. In this region, activation of P2Y_1_ receptors increases renal sympathetic nerve activity, heart rate, and arterial pressure [37]. Astrocytes in RVLM dynamically respond to reductions in brain perfusion pressure, while intracellular pressure rises. The increase in calcium ion concentration triggers a compensatory sympathetic reflex to maintain cerebral blood flow [38].

Reactive astrogliosis is a progressive spectrum—from reversible transcriptional programs and cellular hypertrophy to persistent scar formation and tissue reorganization—and the molecular phenotype of this response is strongly contingent on the initiating insult [39].

### 2.2 Interactions Between Astrocytes and Neuroinflammation

In hypertensive models, the activation of astrocytes is closely linked to the release of inflammatory factors such as IL-1β and TNF-α. These cytokines not only affect astrocyte function but may also exacerbate neuronal injury by increasing BBB permeability. Under hypertensive conditions, astrocyte-mediated neuroinflammatory responses exert multifaceted effects on the structural and functional integrity of the BBB. Activated astrocytes secrete a range of bioactive factors that influence BBB permeability through diverse molecular pathways [40, 41, 42].

Specifically, astrocyte-derived vascular endothelial growth factor (VEGF) is significantly upregulated in the inflammatory microenvironment. This factor activates the endothelial nitric oxide synthase (eNOS)-dependent signaling pathway in vascular endothelial cells, leading to a marked downregulation of key tight junction proteins, including Occludin and Claudin-5. This disruption of protein expression compromises the BBB’s selective permeability, thereby allowing aberrant infiltration of immune cells (such as lymphocytes) into the parenchyma. These events initiate a neuroinflammatory cascade that contributes to subsequent processes such as demyelination [43].

Beyond their pro-inflammatory roles, astrocytes exert protective effects on the BBB through distinct signaling pathways. It is noteworthy that AngII can activate astrocytes through the angiotensin II receptor type 1 (AT1R) receptor, which play a supportive role in the brain. Upon activation, astrocytes increase the production of inflammatory mediators, potentially exacerbating neuroinflammatory responses. However, the AT2R receptor may exert protective or anti-inflammatory effects [44, 45]. Separately, astrocyte-derived all-trans retinoic acid (RA) stabilizes the barrier by activating endothelial retinoic acid receptor beta (RAR-β) signaling, which significantly upregulates key barrier-associated proteins such as vascular endothelial cadherin (VE-cadherin), P-glycoprotein, and zonula occludens-1 (ZO-1) [46].

Hypertension induces significant morphological and functional transformations in astrocytes. A hallmark ultrastructural change observed via electron microscopy is the accumulation of autophagic vacuoles (AVs)—exhibiting features of autophagosomes, lysosomes, and multilamellar bodies—within astrocytic endfeet. This AV accumulation contributes to pathological endfoot swelling and demonstrates a spatial relationship with endothelial tight junctions, suggesting a potential mechanism for the direct anatomical disruption of the BBB and the consequent increase in vascular permeability [41].

In hypertension, astrocytic regulation of the BBB is multifaceted, involving disruptive (VEGF-driven), protective (Ang-1/RA-mediated), and mechanical (autophagic vacuole-induced) pathways. The synergistic interplay of these mechanisms intensifies the CNS inflammatory milieu and propels the pathogenesis of neural injury.

### 2.3 Interactions Between Astrocytes and Vascular Dysfunction

Astrocytes maintain a dynamic and bidirectional relationship with the cerebral vasculature, and this interaction becomes particularly critical under hypertensive conditions. In such states, alterations in astrocytic calcium signaling dynamics may modulate cerebrovascular contractility, particularly in cortical arterioles [47].

Moreover, astrocytes potentiate endfoot Ca^2+^ signaling through Ang II. By activating AT1R, Ang II significantly elevates resting Ca^2+^ levels in astrocyte endfeet and amplifies activity-induced calcium transients. Under physiological conditions, neuronal activity triggers Ca^2+^ signaling in astrocyte endfeet, leading to the release of vasodilatory factors. However, in the presence of Ang II, the endfoot Ca^2+^ signal becomes excessively enhanced, promoting the predominant release of vasoconstrictive factors. This shift converts the vascular response from dilation to constriction. Such alterations may further exacerbate hypertension-associated brain damage [48, 49].

A key finding is the expression of transient receptor potential vanilloid 4 (TRPV4) channels in perivascular astrocytic endfeet, where they sense hemodynamic stimuli (e.g., pressure, flow). In hypertension, both TRPV4 expression and function are augmented, further amplifying the abnormal calcium signal, implicating these channels in mediating pathological astrocytic Ca^2+^ dysregulation. Crucially, the abolition of hypertension-induced parenchymal arteriole hyperconstriction upon TRPV4 blockade or knockout directly demonstrates its essential role in astrocyte-driven neurovascular signaling [50].

Astrocytic involvement in hypertensive vascular remodeling—via regulation of Ang II signaling and TRPV4-mediated Ca^2+^ dysregulation—extends beyond influencing vascular tone to potentially include associated complications like cognitive impairment [48, 51].

**Fig. 1.**
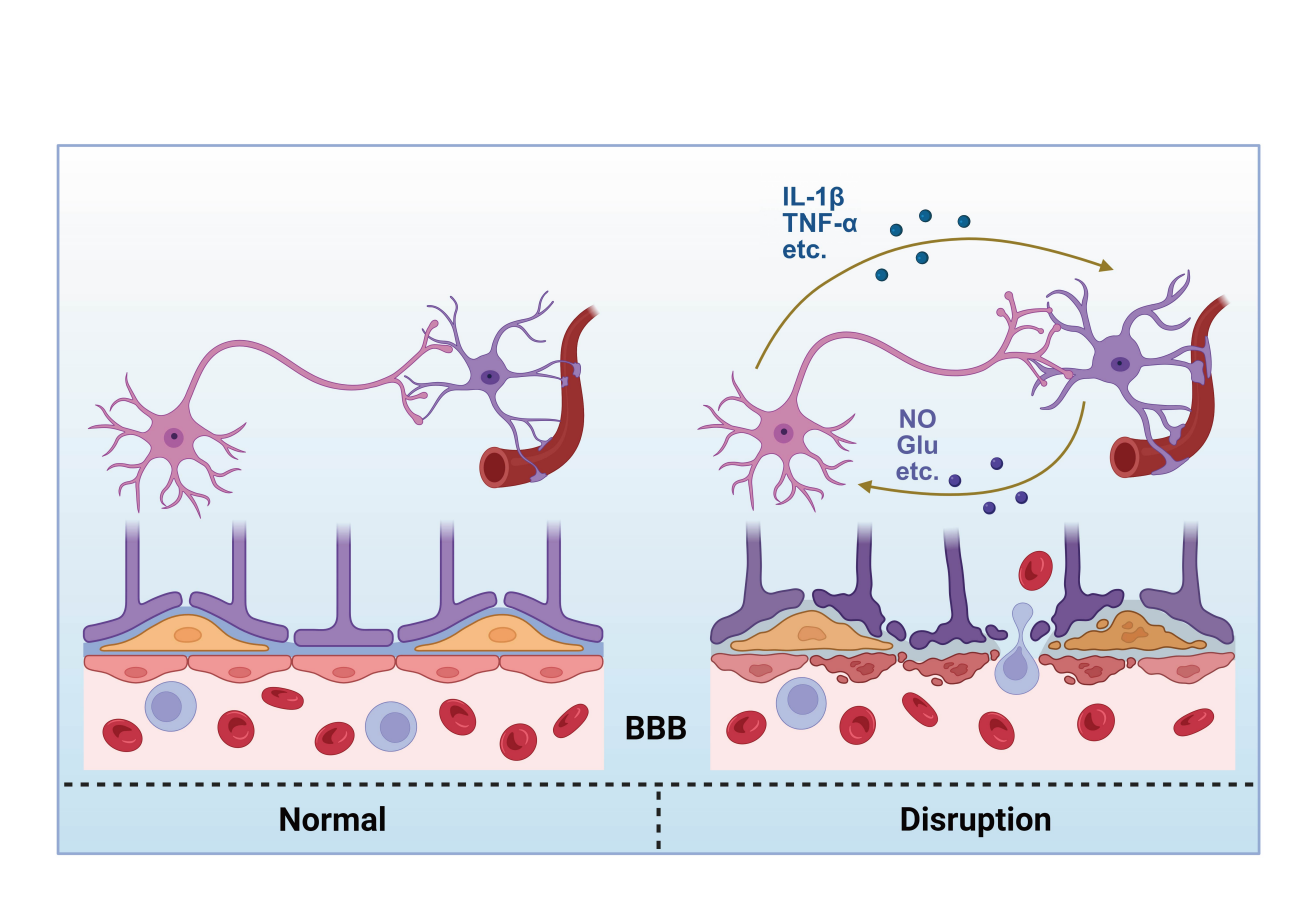
**Schematic diagram of the interaction between astrocytes and neurons mediating blood-brain barrier damage**. BBB, blood–brain barrier; IL-1β, interleukin-1 beta; TNF-α, tumor necrosis factor-alpha; NO, nitric oxide; Glu, glutamic acid. Created with BioRender.com (Toronto, ON, Canada).

In the pathological context of hypertension, astrocytes undergo reactive astrogliosis and neuroinflammatory activation, coupled with vascular dysfunction (Fig. 1). These changes not only compromise astrocytic function but also exacerbate neuronal damage through BBB disruption. Such pathological transformations are closely associated with abnormal neuronal activity. This section examines specific forms of abnormal neuronal activity in hypertension and their influence on blood pressure regulation.

## 3. Neuronal Dysfunction Under Hypertensive Conditions

In hypertension, neuronal dysfunction is predominantly manifested as hyperactivation of the sympathetic nervous system and suppression of parasympathetic [52, 53]. The autonomic imbalance contributes directly to sustained elevations in blood pressure and exacerbates cardiovascular pathology.

### 3.1 Ion Channel Dysregulation in Neurons

In spontaneously hypertensive rats (SHRs), aberrant regulation of neuronal calcium channels-particularly N-type voltage-gated calcium channels-leads to increased intracellular calcium influx, resulting in heightened neuronal excitability. This phenomenon has been linked to disruptions in cyclic nucleotide signaling pathways, including reduced cyclic guanosine monophosphate (cGMP) levels and elevated cyclic adenosine monophosphate (cAMP)–protein kinase A (PKA) activity [54]. These findings suggest that calcium channel dysfunction may serve as a potential early biomarker for sympathetic overactivity in hypertension. Additionally, potassium channel alterations are implicated in the pathogenesis of hypertension. Specifically, downregulation of kir4.1 channel expression and activity contributes to neuronal hyperexcitability, thereby enhancing excitatory output from the RVLM and promoting elevated arterial pressure [55]. The initiation and propagation of action potentials in neurons depend critically on the functional integrity of sodium channels. Under hypertensive conditions, sodium channel dysfunction may disrupt neuronal excitability and interfere with central blood pressure regulatory mechanisms.

### 3.2 Dysregulated Neurotransmitter Release

In hypertensive patients, sympathetic nerve terminals release elevated levels of NE and epinephrine (E). The action of these neurotransmitters on vascular smooth muscle and the heart results in vasoconstriction and a rise in cardiac output, ultimately leading to an increase in blood pressure [56, 57, 58]. Emerging evidence suggests a novel pathway whereby aberrant neurotransmitter release activates the immune system, thereby linking neurogenic dysregulation to exacerbated inflammation and the progression of hypertension. A key underlying mechanism is the ATP-/P2X purinoceptor 7 (P2X7R)-inflammasome axis. In this pathway, neuronally-released ATP serves as a damage-associated molecular pattern (DAMP) that binds P2X7 receptors on innate immune cells, thereby stimulating the NLRP3 inflammasome to generate IL-1β. This process forms a self-reinforcing neuro-immune circuit, sustaining a state of chronic inflammation that contributes to the acceleration of hypertension [27].

### 3.3 Abnormal Neuronal Activity in Neural Populations

#### 3.3.1 Presympathetic PVN Neurons

The PVN is an important higher brain center responsible for integrating cardiovascular, neuroendocrine, and autonomic functions. It regulates sympathetic nerve output by directly or indirectly projecting to the brainstem and spinal cord, primarily by balancing excitatory (e.g., glutamatergic) and inhibitory (e.g., GABAergic) signals to finely control sympathetic activity [59]. In the hypertensive state, the activity of PVN neurons is excessively enhanced, becoming a major source of excitation for sympathetic output. In SHR model, glutamate-mediated synaptic transmission via N-methyl-D-aspartate (NMDA) receptors is enhanced, leading to increased neuronal depolarization and action potentials. Additionally, the phosphorylation of NMDA receptors further amplifies this excitatory signal [60].

#### 3.3.2 NTS Baroreflex Neurons

The NTS is the “first synaptic relay station” for central cardiovascular reflexes, receiving afferent signals from baroreceptors in the carotid sinus, aortic arch, cardiopulmonary receptors, and chemoreceptors. It integrates this information to regulate cardiac output and peripheral vascular resistance. In hypertensive animal models, the sensitivity of the baroreflex is significantly reduced. Prolonged abnormal input from pressure receptors leads to an upward shift in the “set point” of NTS neurons, raising the threshold for their response to elevated blood pressure, thus weakening their inhibitory effect on sympathetic activity [61]. The NTS forms a complex feedback loop with regions such as the PVN, RVLM, and caudal ventrolateral medulla (CVLM) [62]. In the hypertensive state, the inhibitory signals received by the NTS (such as GABAergic projections from CVLM) are reduced, while excitatory inputs (such as glutamatergic projections from PVN) are enhanced, leading to increased firing activity of RVLM sympathetic neurons, which in turn drives blood pressure elevation [63].

#### 3.3.3 Catecholaminergic Neurons

Catecholaminergic neurons are widely distributed in the CNS (such as NTS, RVLM, PVN) and peripheral regions (such as the adrenal medulla), and they regulate cardiovascular function by releasing NE, E, and dopamine. In hypertension, the overall activity of the catecholamine system is increased, leading to excessive activation of the sympathetic nervous system. Medullary A1 neurons (located in CVLM) receive input from pressure receptors and regulate the PVN and anteroventral third ventricle (AV3V) regions, thereby influencing fluid balance and sympathetic output. In hypertension, dysfunction of A1 neurons leads to dysregulation of renal sympathetic nerve activity [64]. Meanwhile, A2 neurons located in the NTS are activated under stress through α2-adrenergic receptors interacting with the renin-angiotensin system (RAS), which in turn promotes an increase in blood pressure [65].

In summary, the abnormal neuronal activity induced by hypertension is not only characterized by ion channel dysfunction and neurotransmitter release imbalance but also accompanied by sustained overactivation of the sympathetic nervous system. However, these neuronal-level changes do not occur in isolation but are closely linked to the functions of glial cells, such as astrocytes. Increasing evidence suggests that astrocytes play a crucial role in maintaining neuronal metabolic homeostasis and clearing excitatory neurotransmitters. When neurons are in a hyperexcitable state, oxidative stress levels are elevated, and inflammation persists, the metabolic and ionic homeostasis regulatory functions of astrocytes are also altered accordingly. Therefore, the abnormal neuronal activity induced by hypertension often interacts with glial cell dysfunction, ultimately forming a “neuronal-glial” pathological feedback loop. Based on this, the following sections will further discuss the specific mechanisms of astrocyte-neuron crosstalk disruption in hypertension, focusing on astrocytic metabolism, glutamate clearance, and the function of Kir4.1 channels.

## 4. Mechanisms of Astrocyte–Neuron Crosstalk Dysfunction in Hypertension

### 4.1 Imbalance in Lactate Metabolism

Under physiological conditions, astrocytes play a pivotal role in supporting neuronal energy metabolism. Upon uptake of neuronally released glutamate, astrocytes enhance glycolysis, producing lactate that is shuttled out via transporters (e.g., MCT1/4). This lactate is then imported into neurons primarily by MCT2, serving as a crucial energy substrate. This metabolic coupling is referred to as the “neuron-glia lactate shuttle” [66]. Specifically, neurons absorb glucose through GLUT3 and metabolize it to generate pyruvate. Although neurons can produce lactate from pyruvate, they predominantly rely on astrocyte-derived lactate, especially during periods of heightened metabolic demand. The neuron-glia lactate shuttle involves a coordinated process whereby astrocytes import glucose via GLUT1, convert it to lactate through glycolysis, and export it via MCT1/4, enabling neurons to import it through MCT2 for use as a primary energy source [67, 68, 69].

#### 4.1.1 Impairment of Lactate Transporters in Hypertension

Hypertension may disrupt this metabolic coupling by downregulating the expression and functional activity of astrocytic lactate transporters (MCT1/4). Evidence suggests that hypertension not only alters neuronal excitability but also induces metabolic reprogramming in astrocytes, leading to reduced lactate production [70]. In parallel, oxidative stress and chronic inflammation associated with hypertension can further impair astrocyte function and attenuate lactate transport efficiency.

#### 4.1.2 Neuronal Energy Deficit and Sympathetic Overactivation

Neurons’ energy stores are reduced when the lactate transporter (MCT1/4) is downregulated because they are unable to get enough lactate to use as an energy source. Although glucose is the primary energy source for neurons, lactate can be a significant alternate energy source in circumstances involving high metabolic demand. However, in hypertensive situations, the energy supply to neurons is inadequate because lactate transfer is blocked, resulting in an energy crisis for the neurons. This crisis may manifest as neuronal dysfunction, increased excitability, and metabolic disturbances [71, 72]. The neuronal energy crisis not only impairs neuronal function locally but may also, by affecting neuronal activity in key brainstem regions such as the RVLM and NTS, lead to enhanced sympathetic nervous system excitability. RVLM neurons regulate sympathetic tone and are sensitive to bioenergetic cues, including intracellular ATP levels, ion channel activity, and glial-derived metabolic support [73]. In hypertension, reduced astrocytic ATP release may impair these regulatory mechanisms, exacerbating RVLM neuron excitability and sympathetic overdrive [74].

#### 4.1.3 M-Current Suppression and Stellate Ganglion Neuron Hyperactivity

During early stages of hypertension, the M-current (a voltage-gated potassium current critical for stabilizing resting membrane potential) is significantly downregulated in stellate ganglion neurons; this downregulation enhances neuronal excitability and contributes to sympathetic hyperactivation [75]. Evidence from SHR models indicates that diminished M-current lowers the depolarization threshold of neurons, which elevates their firing rate and in turn promotes increased sympathetic nerve activity [75]. A striking observation from co-culture studies is that healthy cardiomyocytes can quench hyperexcitability in pathological neurons. This identifies the heart as a potential source of “cardio-to-neural” regulation of sympathetic tone, a compelling concept that demands deeper exploration [76].

### 4.2 Glutamate Clearance Disorder

#### 4.2.1 Glutamate Accumulation and NMDAR Overactivation

Astrocytes play a critical role in regulating extracellular glutamate levels within the CNS by facilitating the uptake and clearance of synaptic glutamate. Under physiological conditions, astrocytic glutamate transporters—primarily EAAT—rapidly remove glutamate from the synaptic cleft, preventing excitotoxic accumulation and maintaining synaptic homeostasis [77, 78]. Under pathological conditions, however, astrocyte function is adversely affected by chronic stress, heightened inflammatory factors (e.g., TNF-α and IL-1β) [79], and enhanced oxidative stress [80], resulting in a diminished ability to take up glutamate. Such astrocytic dysfunction results in synaptic glutamate accumulation and the consequent overactivation of neuronal glutamate receptors, specifically the NMDAR. NMDAR is an ionotropic glutamate receptor that is voltage-gated. Glycine co-activation and depolarization are necessary for its activation in addition to glutamate binding. In hypertensive states, impaired astrocytic glutamate uptake elevates synaptic glutamate levels, triggering prolonged NMDAR activation and neuronal hyperexcitability [81, 82, 83].

Beyond enhancing calcium influx, NMDAR activation modulates neuronal membrane potential and excitability [82], initiates downstream signaling cascades involving protein kinase C (PKC) and calcium/calmodulin-dependent protein kinase II (CaMKII) [84], and ultimately influences neuronal activity. In the context of hypertension, prolonged NMDAR overactivation disrupts neuronal electrophysiological balance and alters neurotransmitter release dynamics, promoting excessive sympathetic nervous system activity [85]. These events collectively initiate a cascade of excitotoxic injury and sustained neuronal dysfunction.

#### 4.2.2 Regulatory Role of Piezo1 Channel Activation

The Piezo type mechanosensitive ion channel component 1 (Piezo1) channel, an important mechanosensitive ion channel, participates in various physiological and pathological processes by sensing mechanical stimuli. Studies have shown that activation of the Piezo1 channel can enhance communication between astrocytes and neurons through an NMDA receptor-mediated mechanism. Specifically, pharmacological activation of the Piezo1 channel enhances signaling between astrocytes and neurons in the mouse cortex, a process likely mediated by NMDA receptors. Additionally, it has been found that activation of the Piezo1 channel (e.g., using Yoda1) significantly increases the frequency of spontaneous inhibitory synaptic currents (SICs) in neurons, while the NMDA receptor antagonist D-2-Amino-5-phosphonopentanoic Acid (D-AP5) inhibits this effect, indicating that NMDA receptors play a crucial role in this process [86].

#### 4.2.3 Enhanced Neuronal Activity in the Paraventricular Nucleus

The hypothalamic PVN serves as a critical integrative hub for cardiovascular regulation by modulating sympathetic outflow. In hypertension, PVN neurons (particularly presympathetic populations) exhibit heightened activity, leading to increased sympathetic drive and further exacerbation of hypertensive pathology. Studies have shown that NMDAR activity in the PVN is significantly upregulated under hypertensive conditions, a phenomenon closely linked to elevated levels of inflammatory mediators such as TNF-α and IL-1β. These cytokines potentiate NMDAR activation in PVN neurons, further amplifying their excitability [79, 87, 88]. Additionally, corticotropin-releasing hormone (CRH) neurons within the PVN are regulated by NMDARs. Their overactivation contributes to hyperactivity of the hypothalamic–pituitary–adrenal (HPA) axis, which in turn promotes further sympathetic nervous system excitation and may establish a pathological feedback loop [89].

#### 4.2.4 Functional Alterations in NMDAR Subunits

NMDARs are heteromeric complexes composed of GluN1, GluN2 (A/B), and GluN3 (A/B) subunits. The specific subunit composition governs receptor kinetics, localization, and signaling properties. The GluN2A subunit, in particular, is involved in presynaptic glutamate release regulation and postsynaptic receptor responsiveness [90]. Under hypertensive conditions, expression levels of GluN2A and GluN2B in the PVN are significantly elevated, leading to enhanced NMDAR activity and heightened neuronal excitability [90]. It is important to note that NMDAR function is also critically modulated by its phosphorylation status. For example, CK2-mediated phosphorylation of GluN2B at Ser1480 has been shown to promote GluN2A upregulation, thereby increasing both presynaptic and postsynaptic NMDAR activation [90]. These molecular alterations collectively contribute to PVN neuronal hyperexcitability and further drive the pathogenesis and progression of hypertension.

### 4.3 Downregulation of Kir4.1 Channels

Mostly found in astrocytes, Kir4.1 is essential for buffering excess extracellular K^+^ produced during neuronal activation [91]. Normally, neuronal firing transiently elevates extracellular potassium concentration ([K^+^]_o_), and astrocytes rapidly clear this excess via Kir4.1-mediated uptake to preserve neuronal excitability and prevent hyperexcitability or toxicity [92].

In hypertension, reduced expression or function of Kir4.1 leads to astrocytic depolarization, impairing their ability to buffer K^+^. This impaired clearance causes accumulation of [K^+^]_o_, which prolongs neuronal depolarization and can trigger abnormal neuronal firing patterns. Experimental evidence shows that in the absence of functional Kir4.1 channels, recovery of [K^+^]_o_ following neuronal stimulation is significantly delayed, particularly under sustained activity, exacerbating excitatory signaling [92]. At the metabolic level, dysfunction of Kir4.1 may induce a shift in astrocyte energy metabolism. Tong *et al*. [93] reported that in Huntington’s disease models, loss of Kir4.1 in striatal astrocytes promotes a metabolic switch from glycolysis to fatty acid oxidation. This reprogramming is accompanied by increased lipid peroxidation and ROS production, enhancing neurotoxicity and disease progression. These findings suggest that Kir4.1 not only regulates ionic homeostasis but also influences astrocytic metabolic substrate preference, with downstream effects on energy supply and oxidative stress [93, 94].

The sympathetic nerve output is also impacted by downregulation of Kir4.1 channels in the ventrolateral region of the medulla oblongata. One of the major central locations controlling cardiovascular activity is the RVLM, which is also the main source of increased sympathetic excitability. In hypertensive situations, elevated sympathetic output is caused by increased neuronal excitability in the RVLM, which raises blood pressure. Studies show that downregulation of Kir4.1 channels causes neuronal depolarization in the RVLM, which changes excitability [95]. Moreover, this process is made worse by neuroinflammation and oxidative stress in the RVLM, which further amplifies sympathetic output [96, 97].

**Fig. 2.**
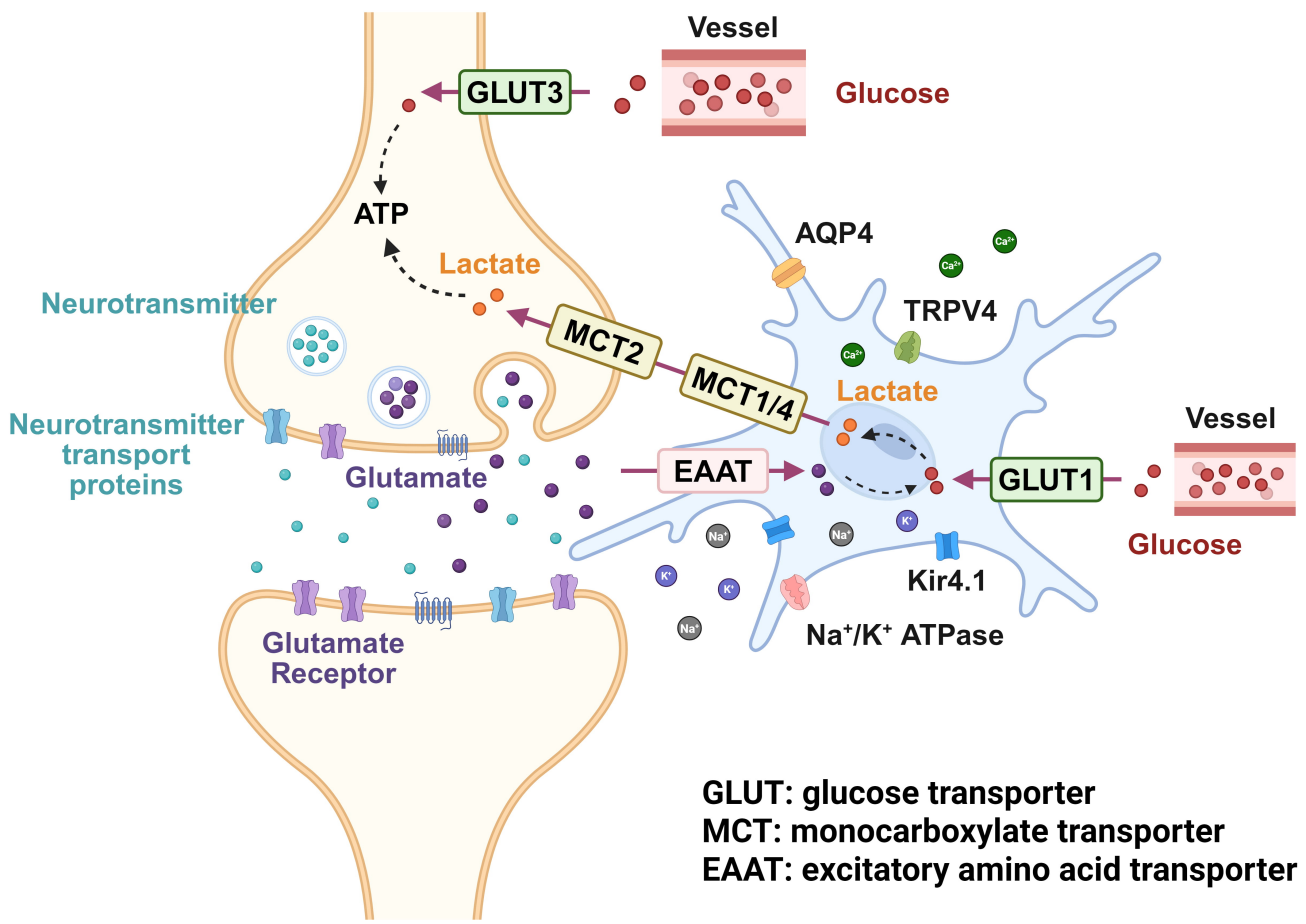
**Mechanism of lactate shuttle, glutamate accumulation, and ion channels in hypertension**. APQ4, aquaporin-4; TRPV4, transient receptor potential vanilloid 4. Created with BioRender.com (Toronto, ON, Canada).

Hypertension induces the interaction between abnormal neuronal activity and astrocyte dysfunction through multiple mechanisms, creating a vicious cycle. Changes such as impaired lactate transport (MCT1/4), decreased glutamate clearance capacity, and downregulation of Kir4.1 channels all contribute to insufficient energy supply to neurons, excessive neuronal excitability, and overactivation of the sympathetic nervous system. Lactate transport impairment prevents neurons from acquiring adequate energy, while excessive activation of NMDARs leads to increased neuronal excitability, and weakened Kir4.1 channel function exacerbates ionic homeostasis disruption (Fig. 2). These pathological changes mutually reinforce each other, further accelerating the progression of hypertension. Therefore, exploring interventions targeting astrocyte-neuron interactions may provide new breakthroughs in the treatment of hypertension.

## 5. Interventions Targeting the Interaction Between Astrocytes and Neurons to Improve Hypertension

### 5.1 The Impact of Physical Exercise on Astrocyte–Neuron Interactions

Excessive sympathetic nervous system activity is a hallmark of hypertension [98, 99], particularly evident in younger individuals and those with elevated resting heart rates, a phenomenon well documented in clinical studies. Exercise exerts anti-inflammatory effects via modulation of sympathetic tone and the HPA axis, and can directly lower blood pressure in hypertensive patients [100]. Specifically, exercise training can reduce sympathetic nervous activity and cortisol levels, indirectly inhibiting adrenaline-induced renin release, thereby decreasing the levels of Ang II and enhancing vasodilation [101]. Preliminary results from our laboratory indicate that moderate-to-low-intensity exercise training can lower blood pressure by down-regulating the expression of PVN pre-sympathetic neurons, which in turn inhibits sympathetic nerve activity in SHR rats.

#### 5.1.1 Exercise-Induced Activation and Morphological Remodeling of Astrocytes

Physical exercise induces functional activation of astrocytes, shifting them from a resting to a reactive state [102]. Saur *et al*. [103] reported that exercise stimulates the outgrowth of both primary and secondary astrocytic processes, thereby strengthening physical contacts and functional integration between astrocytes and neurons. In parallel, Wang D and Wang X [104] demonstrated that exercise modulates the expression of key astrocytic transporters such as EAAT, accelerating extracellular glutamate clearance and mitigating excitotoxicity in neurons. The remodeling of astrocytic morphology and function underlies enhanced bidirectional communication within the neuro-glial network.

#### 5.1.2 Anti-Inflammatory and Antioxidant Properties of Exercise in Astrocytes

Exercise exerts robust anti-inflammatory and antioxidant effects, which are essential for preserving astrocytic function. Evidence suggests that regular physical activity suppresses pro-inflammatory cytokine release and reduces α-synuclein expression, thereby attenuating neuroinflammation. Moreover, exercise inhibits neuroinflammatory signaling via downregulation of the Toll-like receptor 2/myeloid differentiation factor 88/nuclear factor-κB (TLR2/MyD88/NF-κB) pathway in both astrocytes and microglia [102, 105, 106]. These actions help preserve astrocytic homeostasis, prevent pathological overactivation, and protect neurons from oxidative damage and neurotoxicity.

#### 5.1.3 Exercise-Driven Neurotrophic Support via Astrocytes

Exercise enhances astrocyte–neuron crosstalk by upregulating neurotrophic factors such as insulin-like growth factor 1 (IGF-1) and brain-derived neurotrophic factor (BDNF), which are essential for dopaminergic neuronal survival and function [107]. Astrocyte-derived IGF-1 has been shown to exert neuroprotective effects by mitigating oxidative stress in neurons. Exercise-induced elevations in IGF-1 engage astrocytic signaling cascades, reinforcing glial–neuronal communication [108, 109]. Furthermore, physical activity increases astrocytic BDNF expression, which promotes hippocampal synaptic plasticity [110]. Through modulation of these neurotrophic pathways, exercise supports neurogenesis, synaptic remodeling, and neuronal survival, thereby enhancing neuroplasticity in various CNS disease models [111].

#### 5.1.4 Neuroprotective Modulation of Astrocytes Through Exercise

Exercise exerts neuroprotective effects by modulating glutamatergic transmission and promoting astrocytic activation. Evidence indicates that physical activity upregulates the density of GFAP-immunoreactive astrocytes and enhances the release of glutamine, a crucial metabolite in the glutamate–glutamine cycle. These adaptations facilitate glutamate recycling and mitigate excitotoxicity, thereby contributing to neuronal protection [112, 113]. Additionally, exercise promotes upregulation of neuronal NMDAR expression, which is associated with improved synaptic plasticity and cognitive function [114]. These findings underscore the pivotal role of astrocytes in mediating the beneficial effects of exercise on neuronal health and crosstalk.

#### 5.1.5 Long-Term Effects of Exercise on Astrocyte–Neuron Crosstalk

The benefits of exercise are dual-phase: it induces immediate enhancements in astrocyte-mediated support of neurons by rapidly fine-tuning their morphology and activity, while concurrently promoting lasting, pro-plasticity changes that underpin long-term neurological health. Exercise promotes functional motor recovery by enhancing the rate of newborn cell production, their neuronal differentiation, and their successful integration into existing circuits—all of which are processes critical to functional motor outcomes [115]. Particularly, balance and coordination exercises stimulate synaptic remodeling and cerebral angiogenesis [116, 117]. These long-term effects indicate that exercise not only transiently enhances astrocyte–neuron interactions but also contributes to long-term adaptability and functional resilience of the CNS.

### 5.2 Effects of Antihypertensive Agents on Astrocyte and Neuron Function

#### 5.2.1 ACE Inhibitors

Angiotensin-converting enzyme (ACE) inhibitors, widely used in hypertension management, lower blood pressure by inhibiting ACE activity and reducing Ang II production, thereby attenuating vasoconstriction [118]. Importantly, ACE is also expressed in the brain, where it modulates astrocytic function. Inhibiting central ACE activity has been shown to reduce astrocytic Ang II levels, thereby mitigating neuroinflammation and oxidative stress and preserving neuronal integrity [118].

#### 5.2.2 Anti-Inflammatory Agents

Neuroinflammation, driven by activated microglia and astrocytes, contributes to increased sympathetic tone in hypertension via the release of pro-inflammatory cytokines such as IL-1β, IL-6, and TNF-α [119]. Anti-inflammatory drugs like minocycline can attenuate this response by suppressing microglial activation and reducing cytokine release. Additionally, overexpression of IL-10, an anti-inflammatory cytokine, mimics the blood pressure–lowering effects of minocycline, suggesting a potential immunomodulatory strategy for hypertension control [119].

#### 5.2.3 Calcium Channel Blockers

Calcium channel blockers inhibit calcium ion influx by blocking calcium channels on vascular smooth muscle cells, thereby reducing smooth muscle contraction, dilating blood vessels, and lowering blood pressure [120]. However, by controlling the calcium signal transduction in astrocytes, calcium channel blockers may potentially have an impact on neuronal function. For example, astrocytes’ calcium signal transduction controls glutamate release and metabolism, which affects neuronal excitability [121]. Therefore, calcium channel blockers may help lower blood pressure by modulating the function of astrocytes, inhibiting sympathetic nervous activity and excessive neuronal excitability.

#### 5.2.4 Antioxidants

A key mechanism contributing to hypertension is oxidative stress. In hypertension, astrocytes and neurons experience oxidative stress, resulting in ROS accumulation and consequent cellular impairment. Antioxidants protect these cells by neutralizing free radicals to reduce oxidative damage [122].

#### 5.2.5 Emerging Therapeutics

Novel agents are being developed to target astrocyte–neuron crosstalk in the context of hypertension. For example, medicinal oligosaccharides (MOOs) have been shown to upregulate mitofusin-2 (Mfn2) and activate the PI3K/Akt/mTOR pathway to promote mitophagy, thereby facilitating the clearance of damaged mitochondria in astrocytes. This alleviates oxidative stress and neuroinflammation, improves astrocyte function, and may reduce hypertension-associated depressive symptoms [123].

## 6. Conclusion

Central to the pathophysiology of hypertension is the dysregulation of astrocyte-neuronal crosstalk. Our analysis concludes that the loss of homeostatic balance in this critical communication is a pivotal etiological factor. Driven by genetic predispositions, environmental exposures, and lifestyle choices, this imbalance activates a cascade of pathological mechanisms that promote sustained blood pressure elevation and its associated cardiovascular complications.

Current research redefines hypertension as a disease of the central “metabolic-neuro” network, where astrocytes are critical. Their failure to maintain homeostasis (evidenced by lactate metabolic blocks, impaired glutamate uptake, and reduced Kir4.1 activity) precipitates regional neuronal energy deficits and hyperexcitability, culminating in sympathetic-driven hypertension. This paradigm shift from peripheral origins highlights the need to explore individual differences in astrocyte-neuron dialogue, paving the way for precise, mechanism-based interventions and improved prevention.

In conclusion, dysregulated astrocyte-neuron communication is a key factor of hypertension. Elucidating its detailed mechanisms is the gateway to effective therapy, and we look forward to innovative research that transforms this promise into clinical reality.

## Abbreviations

AVs, autophagic vacuoles; ACE, angiotensin-converting enzyme; Ang I, angiotensin I; Ang II, angiotensin II; ATP, adenosine triphosphate; BBB, blood-brain barrier; BDNF, brain-derived neurotrophic factor; CX43, connexin 43; CRH, corticotropin-releasing hormone; CNS, central nervous system; CVLM, caudal ventrolateral medulla; CNTF, ciliary neurotrophic factor; E, epinephrine; eNOS, endothelial nitric oxide synthase; GFAP, glial fibrillary acidic protein; GABA, gamma-aminobutyric acid; HSPs, heat shock proteins; IL-1α, interleukin-1 alpha; IL-1β, interleukin-1 beta; IL-6, interleukin-6; IGF-1, insulin-like growth factor-1; NO, nitric oxide; NE, norepinephrine; NTS, nucleus of the solitary Tract; NMDA, N-Methyl-D-Aspartate; PVN, paraventricular nucleus; RA, retinoic acid; RAR-β, retinoic acid receptor beta; ROS, reactive oxygen species; RAS, renin-angiotensin system; RVLM, rostral ventrolateral medulla; SHR, spontaneously hypertensive rat; TNF-α, tumor necrosis factor-alpha; TGFβ, transforming growth factor-beta; VEGF, vascular endothelial growth factor; 5-HT, 5-hydroxytryptamine.
